# A calculator for musculoskeletal injuries prediction in surgeons: a machine learning approach

**DOI:** 10.1007/s00464-024-11237-4

**Published:** 2024-09-16

**Authors:** Luis Sánchez-Guillén, Carlos Lozano-Quijada, Álvaro Soler-Silva, Sergio Hernández-Sánchez, Xavier Barber, José V. Toledo-Marhuenda, Francisco López-Rodríguez-Arias, Emilio J. Poveda-Pagán, César González Mora, Antonio Arroyo

**Affiliations:** 1 General and Gastrointestinal Surgery Department, University General Hospital of Elche, Miguel Hernández University, 11 Almazara Street, 03203 Elche, Alicante, Spain; 2 Physiotherapy, Pathology and Surgery Department, Translational Research Center, INTRAFIS Research Group, Miguel Hernández University of Elche, Avenue of the University of Elx, S/N, 03202 Elche, Alicante, Spain; 3 Operations Research Center, Miguel Hernández University of Elche, Avenue of the University of Elx, S/N, 03202 Elche, Alicante, Spain; 4 Department of Computer Science, University of Alicante, San Vicente del Raspeig Street, S/N, 03690 Alicante, Spain

**Keywords:** Ergonomics, Musculoskeletal pain, Risk assessment, Surgeons, Surgical procedures, Self-care

## Abstract

**Background:**

Surgical specialists experience significant musculoskeletal strain as a consequence of their profession, a domain within the healthcare system often recognized for the pronounced impact of such issues. The aim of this study is to calculate the risk of presenting musculoskeletal injuries in surgeons after surgical practice.

**Methods:**

Cross-sectional study carried out using an online form (12/2021–03/2022) aimed at members of the Spanish Association of Surgeons. Demographic variables on physical and professional activity were recorded, as well as musculoskeletal pain (MSP) associated with surgical activity. Univariate and multivariate analysis were conducted to identify risk factors associated with the development of MSP based on personalized surgical activity. To achieve this, a risk algorithm was computed and an online machine learning calculator was created to predict them. Physiotherapeutic recommendations were generated to address and alleviate each MSP.

**Results:**

A total of 651 surgeons (112 trainees, 539 specialists). 90.6% reported MSP related to surgical practice, 60% needed any therapeutic measure and 11.7% required a medical leave. In the long term, MSP was most common in the cervical and lumbar regions (52.4, 58.5%, respectively). Statistically significant risk factors (OR CI 95%) were for trunk pain, long interventions without breaks (3.02, 1.65–5.54). Obesity, indicated by BMI, to lumbar pain (4.36, 1.84–12.1), while an inappropriate laparoscopic screen location was associated with cervical and trunk pain (1.95, 1.28–2.98 and 2.16, 1.37–3.44, respectively). A predictive model and an online calculator were developed to assess MSP risk. Furthermore, a need for enhanced ergonomics training was identified by 89.6% of surgeons.

**Conclusions:**

The prevalence of MSP among surgeons is a prevalent but often overlooked health concern. Implementing a risk calculator could enable tailored prevention strategies, addressing modifiable factors like ergonomics.

Surgical specialists are greatly affected at the musculoskeletal level by their work, being described as one of the areas most affected by this issue in the healthcare system [1, 2]. It should come as no surprise that surgeons are in this group, due to repetitive movements, long periods of standing, bending around the patient and continuous loading on specific muscle groups. A study by Park et al. found that up to 87% of surgeons performing minimally invasive surgery experienced work-related pain [1, 3]. This can negatively affect their lives in particular and the healthcare system in general by accelerating surgeons’ retirement [4].

The prevalence of work-related musculoskeletal disorders among surgeons is reported to range from 20 to 70% [5–7]. This disorder most commonly occurs in the neck, back and shoulders, and lack of ergonomic setup and poor posture are cited as underlying culprits [8–12], with prevalence rates of neck pain as high as 80% among surgeons in Europe [13] and in Hong Kong [2, 12]. Several groups have studied the consequences of implementing ergonomics and microbreak in clinical practice and reported a benefit in prolonged surgery and in long-term on Surgeons’ Health [14–18].

The purpose of this study is to assess the national prevalence and practice-associated musculoskeletal pain and musculoskeletal disorders among surgeons and to identify risk factors that might predict these pathologies. Afterwards, and based on these results, to devise a personalized prevention strategy.

## Material and methods

A closed electronic survey based on an extended version [19] of the Nordic Musculoskeletal Questionnaire [20] was sent via google forms to members of the Spanish Association of Surgeons (AEC). This anonymous survey contained 59 items and was opened between December 2021 and March 2022. Surgeons were classified according to their training into trainees and specialists and according to their work experience into < 10 years (early career), 10–20 years (mid-career) and > 20 years (senior) (According to Super’s et. al.) [21]. The data requested in the survey were: demographic data, data on operating room activity and physical activity, on pain (presence or absence of pain related to operating room activity, history of pain, pain intensity and duration) as well as ergonomic knowledge, practices and suggestions. All data obtained were filtered, reviewed and subjected to statistical analysis for the extraction of results.

To assess risk and provide appropriate recommendations, machine learning techniques, such as Random Forests and Gradient Boosting, were employed to address the complexity and non-linear nature of our data. The most influential predictors were effectively selected to assess the impact of variables on model prediction error, guiding the variable selection process and ensuring the inclusion of significant predictors in injury risk evaluation. Subsequently, a logistic regression model was constructed using the selected predictors to estimate the probability of injury occurrence. The beta coefficients of the logistic regression model were leveraged to quantify the influence of each predictor on the outcome, thereby optimizing the accuracy and reliability of our risk probability calculations for real-world applications. The online calculator was created using HTML, CSS, and JavaScript technologies, adhering to W3C standards to ensure accessibility and compatibility with all devices and web browsers. Its primary purpose is to assess subject-specific risk, assisting in the selection of ergonomic recommendations provided by physiotherapists. The study protocol was approved by The Ethics and Experimental Research Committee of Miguel Hernandez University (DPC-SHS-01.21). All of the participants were informed of the study objectives and signed an electronic informed consent form for voluntary participation.

## Results

### Surgeons characteristics

A total of 2058 surveys were sent out to surgeons, with a 31.6% response rate (651 responses). Of the respondents, 112 were trainees, and 539 were specialists The specialists were further categorized as 167 early career, 130 mid-career, and 242 senior trainee participation rates by year of training (from year 1 to 5) were as follows: 16% (18 trainees), 18.8% (21). trainees), 29.5% (33 trainees), 14.3% (16 trainees), and 21.4% (24 trainees). The median age of all participants was 42 years, with a range of 33–53 years Most of the participating surgeons. 58.7%) were female. Among all survey respondents, 90.6% reported experiencing some form of musculoskeletal pain (MSP) related to their surgical practice. The most frequently used technique among surgeons was laparoscopic surgery, performing more than 50 procedures per year in 45.6% of the cases. 84% of the respondents performed 17–24 h on-call shifts, with 91.4% (498) reporting MSP; however, 88.6% of the 106 surgeons who did not perform on-call shifts also reported some MSP in the last 12 months. Surgical and MSP data for the whole surgeon’s cohort are summarized in Table 1.

**Table 1:** Surgeons and ailments details for the whole group of surgeons

Variable	N (%)
Professional profile	
Trainee	112 (17.2)
Specialist early career	167 (25.6)
Specialist mid-career	130 (20.0)
Specialist senior	242 (37.2)
Type of hospital	
Public	492 (75.8)
Private	31 (4.8)
Both	126 (19.4)
Gender	
Female	382 (58.7)
Male	269 (41.3)
Surgery type (performed > 50 procedures/year)	
Open surgery	250 (38.8)
Laparoscopic surgery	297 (46.4)
Minor surgery	239 (31.4)
Endoscopic surgery	16 (3.5)
Robotic surgery	3 (0.7)
Ailment /pathology due to surgeries in the last 12 months	
Cervical	341 (52.4)
Lumbar	381 (58.5)
Shoulder’s tendinopathy	149 (22.9)
Epicondylitis	77 (11.8)
Quervain’s tendinopathy	37 (5.7)
Carpal tunnel syndrome	30 (4.6)
Biceps tendinopathy	20 (3.1)
Frozen shoulder	16 (2.5)
Having required treatment/measures to palliate pain	
Pharmacological	280 (43)
Physiotherapy	275 (42.2)
Orthopedic	52 (8)
Surgical	16 (2.5)
Any measure	392 (59.8)
Have lost days of work due to MSP	76 (11.7)

*MSP* Musculoskeletal pain

**Fig. 1:**
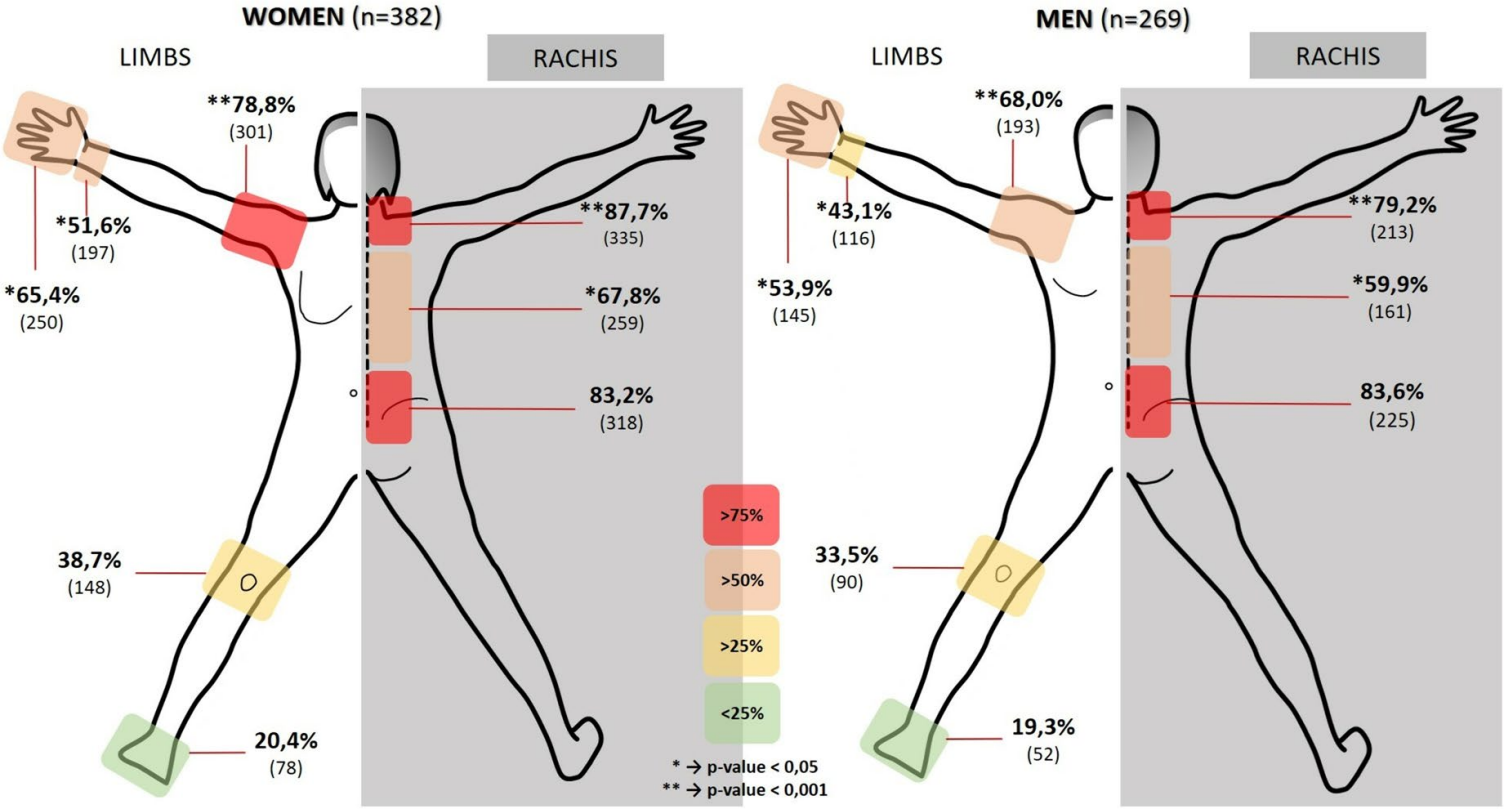
Percentage of pain/discomfort presence by anatomical regions and by gender

**Fig. 2:**
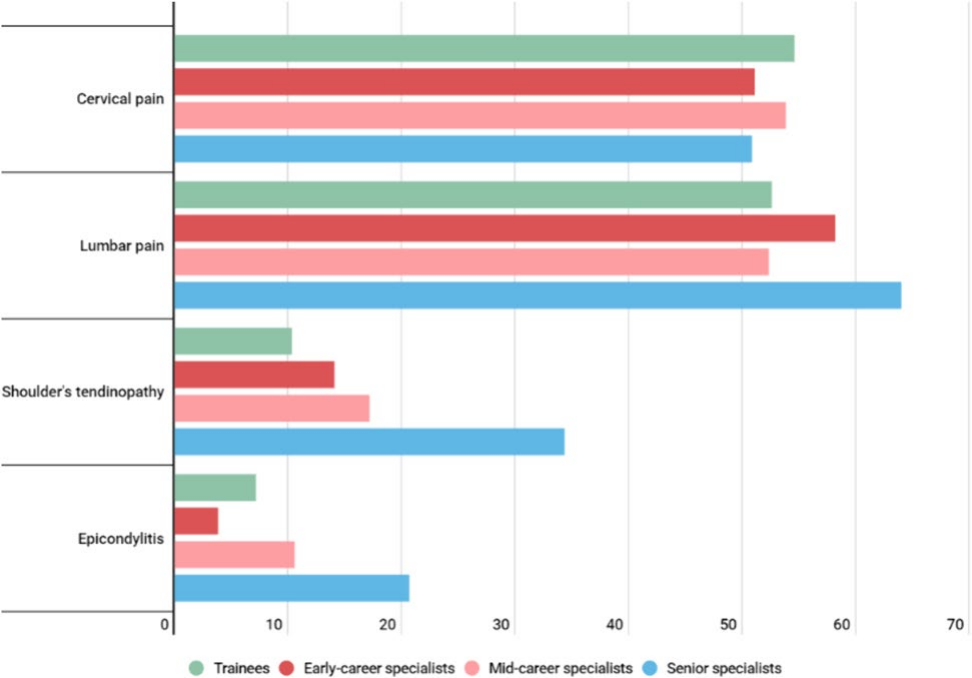
Percentage of referred musculoskeletal pain among different groups of surgeons

The most prevalent MSP reported occurred in the cervical and lumbar regions, both in the short-term (hours) with rates of 19.2 and 25%, respectively, and in the long-term (year) with rates of 52.4 and 58.5%, respectively. Figure 1 represents the percentages of pain in each of the anatomical areas analyzed, differentiated by sex. The frequencies of MSPs obtained different results when classified by training and work experience (Fig. 2). Of the 90.6% (591) of the surgeons surveyed who reported having suffered an MSP in the last 12 months, 90.1% of the specialists and 94.7% of trainees. The frequency of the requirement of any therapeutic measure (pharmacological, physiotherapeutic, orthopedic or surgical) was 60% (390). Regarding the question of whether surgeons have missed days of work due to MSP, 76 surgeons (11.7%) responded affirmatively.

The rate of knowledge of ergonomics recommendations was 11.6% in trainees and 25.4% between consultants. More than 75% of both groups put them into practice after training mainly intraoperative stretching 33.3% (217), use of stools 29.8% (194), 6.4% (42) as mini-breaks, 1.1% (7) as standing pads and 0.6% (4) as compression stockings. The source of information was in decreasing order: word of mouth (10.8% (70)); internet (9.5% (62)); courses or congresses (6.5% (42)) and residency (3.7% (24)). 89.6% (577) of the surgeons surveyed considered that the training of medical students or surgical residents in this field was insufficient and 93.6% (613) recommended implementing recommendations in ergonomics and posture.

### Risk factors

The variables associated with MSP in the multivariate analysis are shown in Table 2, where the risk (OR CI 95%) of variables to the most frequent MSP with statistically significant (*p* < 0.05) values were for trunk pain long intervention without breaks and inappropriate location of the laparoscopic screen (3.02, 1.65–5.54 and 2.16, 1.37–3.44, respectively); for lumbar pain, obesity BMI (4.36, 1.84–12.1); and for cervical pain, inappropriate location of the laparoscopic screen (1.95, 1.28–2.98).

**Table 2:** Representation of the odds ratio values with its confidence interval CI 95% for the different variables implied in the musculoskeletal risk computation, for each body area

PREDICTORS		PAIN LOCATION							
		Body trunk	Lumbar	Shoulders	Dorsal	Hands	Knees	Foot/ankle	Neck
Age, OR (CI 95%)	**–**	**–**	**–**	**–**	0.96 (0.93**–**0.99)*	0.97 (0.95**–**0.99)*	**–**	0.8 (0.27**–**2.44)	0.98 (0.96**–**1)
Gender, OR (CI 95%)	Male	0.28 (0.14**–**0.53)*	**–**	**–**	0.03 (0.00**–**0.34)*	**–**	1.78 (0.88**–**3.62)	**–**	0.56 (0.36**–**0.86)*
	Female	1	**–**	**–**	1	**–**	1	**–**	1
BMI, OR (CI 95%)	< 25	**–**	1	**–**	**–**	**–**	1	1	**–**
	25–30	**–**	1.06 (0.66**–**1.71)	**–**	**–**	**–**	1.46 (0.79**–**2.72)	1.45 (0.75**–**2.73)	**–**
	> 30	**–**	4.36 (1.84**–**12.12)*	**–**	**–**	**–**	5.56 (1.61**–**25.77)*	3.97 (1.58**–**9.85)*	**–**
Height, OR (CI 95%)	**–**	1.04 (1.01**–**1.08)*	**–**	**–**	**–**	**–**	**–**	**–**	**–**
Total surgeries per year, OR (CI 95%)	< 25	**–**	**–**	1	**–**	**–**	**–**	**–**	**–**
	25–50 vs < 25	**–**	**–**	1.52 (0.74**–**3.21)	**–**	**–**	**–**	**–**	**–**
	> 50	**–**	**–**	2.19 (1.07**–**4.58)*	**–**	**–**	**–**	**–**	**–**
Perform high frequency of surgery (3–5 per week), OR (CI 95%)	Yes	**–**	**–**	1.6 (1.02**–**2.53)*	**–**	**–**	1.55 (0.95**–**2.55)	**–**	**–**
	No	**–**	**–**	1	**–**	**–**	1	**–**	**–**
Duration of the surgery, OR (CI 95%)	Short	1	**–**	**–**	**–**	**–**	1	**–**	**–**
	Long	2.25 (1.14**–**4.43)*	**–**	**–**	**–**	**–**	2.37 (1.17**–**5.25)*	**–**	**–**
Type of surgery performed, OR (CI 95%)	Not MIS	1.71 (0.91**–**3.19)	1.83 (1.02**–**3.29)*	**–**	**–**	**–**	**–**	**–**	**–**
	MIS	1	1	**–**	**–**	**–**	**–**	**–**	**–**
Minor surgery procedures per year, OR (CI 95%)	< 25	1	**–**	**–**	1	**–**	**–**	**–**	**–**
	25–50	1.38 (0.82**–**2.35)	**–**	**–**	1.75 (0.98**–**3.16)	**–**	**–**	**–**	**–**
	> 50	2.08 (1.21**–**3.63)*	**–**	**–**	2.03 (1.13**–**3.71)*	**–**	**–**	**–**	**–**
Performing endoscopic surgery procedure, OR (CI 95%)	Yes	0.62 (0.37**–**1.01)	**–**	**–**	0.29 (0.14**–**0.57)*	1.66 (1.02**–**2.72)*	**–**	**–**	**–**
	No	1	**–**	**–**	1	1	**–**	**–**	**–**
Instrumental bad ergonomics, OR (CI 95%**)**	Yes	**–**	**–**	**–**	**–**	2.29 (1.48**–**3.56)*	**–**	**–**	**–**
	No	**–**	**–**	**–**	**–**	1	**–**	**–**	**–**
Inappropriate location of the laparoscopic screen, OR (CI 95%)	Yes	2.16 (1.37**–**3.44)*	**–**	2.69 (1.21**–**6.44)*	2.51 (1.57**–**4.05)*	**–**	1.53 (1.05**–**2.22)*	1.64 (1.52**–**4.70)*	1.95 (1.28**–**2.98)*
	No	1	**–**	1	1	**–**	1	1	1
Long interventions without breaks, OR (CI 95%)	Yes	3.02 (1.65**–**5.54)*	1.93 (1.04**–**3.62)*	**–**	**–**	**–**	**–**	**–**	**–**
	No	1	1	**–**	**–**	**–**	**–**	**–**	**–**

Marked with an asterisk, the statistically significant values (IC 95% value 1 for OR excluded)

*MIS* minor invasive surgery, *BMI* body mass index

### Model performance and calculator creation

**Fig. 3:**
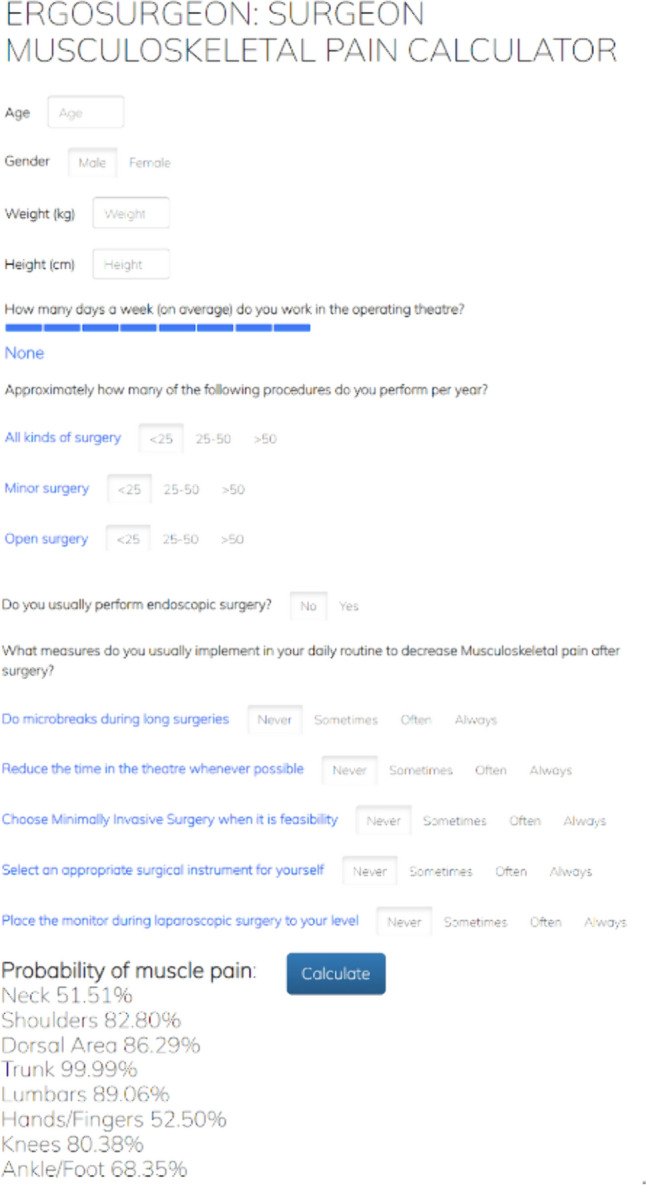
Screenshot of the calculator website, showing the functioning of the risk evaluation

Sensitivity, specificity, predictive values and accuracy of the predictive model were described (Table 3). Based on these results, an online calculator was developed to evaluate the risk of MSP (Fig. 3). By inserting the surgeon characteristics and number of operations, a score is assigned to each variable (which corresponds to the percentile). The sum of these scores returns an overall result indicating the probability of post-operative MSP (https://surgeonpaincalculator.000webhostapp.com/) [1–4].

**Table 3:** Statistical calculations for the predictive model in null-mild vs moderate-severe pain

Statistical parameters of the model	Body area							
	Body trunk	Neck	Shoulders	Dorsal	Lumbar	Hands	Knees	Feet and ankles
Accuracy	0.738	0.670	0.615	0.577	0.732	0.667	0.600	0.786
(95% CI)	(0.662, 0.803)	(0.567, 0.762)	(0.509, 0.712)	(0.479, 0.669)	(0.640, 0.811)	(0.525, 0.789)	(0.459, 0.729)	(0.725, 0.838)
P-value [Acc > NIR]	0.694	0.025	0.041	0.389	0.382	0.020	0.558	0.347
Mcnemar’s P-value	0.165	0.377	0.021	0.000	0.201	0.010	0.0002	3.247e-11
Sensitivity	0.469	0.548	0.435	0.823	0.406	0.423	0.939	1.000
Specificity	0.856	0.764	0.780	0.265	0.863	0.893	0.091	0.061
PPV	0.590	0.639	0.645	0.586	0.542	0.786	0.608	0.783
NPV	0.785	0.689	0.600	0.542	0.784	0.625	0.500	1.000
Prevalence	0.306	0.433	0.479	0.559	0.286	0.482	0.600	0.772
Detection rate	0.144	0.237	0.208	0.460	0.116	0.204	0.564	0.772
Detection prevalence	0.244	0.371	0.323	0.784	0.214	0.259	0.927	0.986
Balanced Accuracy	0.663	0.656	0.607	0.544	0.634	0.658	0.515	0.531

Body trunk category contains the neck, shoulders, dorsal and lumbar areas

*PPV* positive predictive value, *NPV* negative predictive value, *CI* confidence interval, *ACC* accuracy, *NIR* no information rate

## Discussion

In the present study, the prognosticators of musculoskeletal pain between surgeons after surgery were assessed. Multivariable analysis showed that several surgeon-related variables (age, height, gender, BMI) and surgery-related variables (number of minor and total surgeries performed per year, high frequency surgery performance (3–5 per week), inappropriate location of laparoscopic screens, duration and type of surgery performed, consecutive hours of surgery without breaks and instrumental bad ergonomics) were independent risk factors. We developed a useful calculator incorporating these factors, which can be used to predict the risk for MSP for each given surgeon.

Despite the advances in surgery and the technological implementation in operating rooms, operating rooms are still not designed ergonomically nor with the surgeon’s health in mind and therefore, prevention of MSP among surgeons still represents a gray area in the Health System [22]. MSP and its complications significantly affect human and economic resources, sick leave and costs. Since this is a sector with a high impact on healthcare performance, surgeons must be evaluated and advised on the correct implementation of postural hygiene and ergonomics measures during surgical procedures [1, 23].

After studying the different variables that influence the total risk of developing musculoskeletal pain, several factors that have a special effect can serve as predictors of risk. Age has been found to be a protective factor, especially in feet and ankles, back, hands and neck. Similar findings have been reported previously for the age factor [23, 24]. This is because age is one of the determining factors for the appearance of musculoskeletal pain in the general population [25, 26]. Especially in surgeons, and also with statistical significance in our study, performing surgeries with a high frequency (3–5 times per week) has been shown to be a risk factor. Muscle fatigue and lack of recovery periods after excessive loading of certain muscle groups has been highly described [27, 28]. Laparoscopic instruments were described as one of the main contributing factors to the origin of the symptoms, maybe the differences in hand size, hand dominance and other factors between surgeons be the underlying cause of this [29, 30]. This observation is consistent with the high rate of hand, fingers and wrists pain reported. It has also been described how the laparoscopic instruments require almost six time greater the force for their support than the required in open surgery, losing the use of force at the tip of the instruments, especially accentuated by the effect produced by the intracorporeal aspect of the instrument moving in the opposite direction of the surgeons hand when maneuvering [22]. Likewise, many subjects related the use of other surgical accessories (front light, magnifying glasses, and microscopes) with these musculoskeletal pains [31].

We have also found differences between women and men in the perception of pain or discomfort in the operating room. One of the reasons could be a physiological difference between women and men in pain perception [32]. In the population studied, other factors should be taken into account, such as the distribution by experience groups and the generational change by sex that is occurring among surgeons [33]. Furthermore, the possibility of adaptation according to height to the operating room or the size standards of the surgical instruments themselves are factors that can influence the physical characteristics of male and female surgeons [29, 34].

According to the survey’s responses, a large portion of surgeons not only present pain of alarming intensity for the care practice associated with their work, but also these pains are often maintained over time, affecting their personal lives as well. Despite the high prevalence of these ailments, their reporting rate is relatively low, and most of the time the surgeon has to continue with his or her work despite these conditions, with the impact this can have on patient care.

One of the relevant findings in this study is the difference in surgical injuries between residents and experienced surgeons, being greater in the first case. In addition, it has been observed that age is a protective factor against pain and injuries associated with surgical practice. This observation was previously described by other groups [24, 35]. Possibly the acquisition of experience and safety, as well as the adequate preparation of the equipment are the key to explain these differences. It is important to note at this point that normally the equipment in surgery is oriented to the main surgeon who is usually mid-career, this not being the case for the support surgeon.

It should be noted that a large part of the respondents required assistance (60%, *n* = 390) (pharmacological, physiotherapy, orthopedic, or surgery) to alleviate pain. These findings expose the real impact that these conditions have on the personal and care activity of these professionals. Furthermore, this observation is supported by the statement of 11.7% (*n* = 76) of the surgeons surveyed, of having lost days of work due to musculoskeletal problems.

Also relevant is the consensus among surgeons regarding the lack of training in ergonomics and good postural hygiene to carry out their work. Additionally, these responses are supported by the low rate of use of measures to avoid muscle load and injuries, such as the use of stools, microbreaks, or postural changes, which in other studies have been found to be vital to prevent this type of injury. Other authors have described previously how the implantation of ergonomic measures, training of specialists, and teaching through videos and infographics can decrease the risk of developing musculoskeletal injuries in these subjects [8, 23]. According to this observation, we establish a series of ergonomic recommendations to share with the surgeon once their risk has been calculated, this can help the professional to implement better postures and reduce musculoskeletal damage in the long term. It has also been reported that the implementation of microbreaks has a positive impact on surgeons’ musculoskeletal pain, stress release and fatigue [3], although this observation is not as clear for short surgeries and in the short term [18]. Some of the measures such as the implementation of microbreaks may be challenging due the attitudes and beliefs of some surgeons. It may be perceived that microbreaks is a trivial measure that enlarges the time of surgery and limits the number of operations. This reasoning is supported by the findings of Engelman et al. [15] that reported surgeons that considered themselves to be fast, rated the brakes lower than those considered slower [1].

When it comes to estimate the implementation of ergonomic measures in organizations, costs are reported to be a limiting factor, as their rentability depends ultimately on the benefits obtained afterwards. In the light of this, there is an emerging trend to perform cost–benefit analyses (CBA) with a safety and productivity enhancing purpose, especially in the field of implementation of ergonomic measures for safety and health [36]. The development of CBA models [37, 38] can allow the implementation of a cost–benefit calculator and optimization of the associated procedures and costs. A very interesting alternative to this approach is the development of a risk calculator, once the risk factors have been identified, to formulate and implement the appropriate measures and recommendations. That is why the developed calculator can allow individual surgeons, surgical services and health systems to predict MSP and develop measures that allow its measurement.

It is important to take these results with caution and to take into consideration the following detected limitations: The response rate of 31.6% could introduce selection bias, overestimating the prevalence of musculoskeletal pain. This low response rate is common in voluntary population surveys, where those who have experienced the condition in question are more likely to participate. Additionally, data collection using self-report is subject to recall bias. Participants may not accurately recall or report their pain experiences, which could affect the reliability of the results. It is important to consider these limitations when interpreting the findings of the study and generalizing the conclusions to the target population. These limitations could be corrected with future studies using methodologies that minimize these biases, such as longitudinal surveys or case–control designs.

The promising advance and implementation of robotic surgery seems to be a healthier and safer alternative for the surgeon’s health, possibly displacing the traditional technique. Although this new alternative still causes muscle pain in surgeons, these professionals apparently develop less musculoskeletal pain in all body areas [36, 39] and less mental fatigue for the surgeon [40]. However, longer preparation time and sterile drapes, and an increase in total operating time due to the slow movement of the robot’s careful arms, have been reported against this technique [41]. The most concerning physical symptoms reported by robotic surgery procedure include finger and eye fatigue, and neck stiffness [42, 43], despite all of these observations, there is no consensus in the literature, and many biases are present in the studies to date, according to a meta-analysis [44] the only significant and robust difference in the literature for pain between robotics and traditional surgery was recorded for biceps. Further study of the advantages and disadvantages of this new technique is required, especially in the long-term outcomes.

Despite the tendency to transition towards robotic surgery, it will take a long time to become the standard. In the meantime, it is necessary to raise awareness among surgeons about the ergonomics, such as stretching or establishing inter and intraoperative breaks or microbreaks to improve not only their health but also their performance and, consequently, patient service.

In conclusion, the high rate of musculoskeletal pain is a silenced health problem in surgeons. Some non-modifiable factors such as age, type of surgery performed and duration of surgeries were examined as contributors to the onset of MSP. However, other modifiable factors as inappropriate location of laparoscopic screens, consecutive hours of surgery without breaks and instrumental bad ergonomics were also considered in the assessment. The use of a risk calculator could aid in the evaluation and implementation of a personalized prevention strategy minimizing MSPs in the surgical setting.
